# Predictive Analytics for Care and Management of Patients With Acute Diseases: Deep Learning–Based Method to Predict Crucial Complication Phenotypes

**DOI:** 10.2196/18372

**Published:** 2021-02-12

**Authors:** Jessica Qiuhua Sheng, Paul Jen-Hwa Hu, Xiao Liu, Ting-Shuo Huang, Yu Hsien Chen

**Affiliations:** 1 Department of Operations and Information Systems David Eccles School of Business University of Utah Salt Lake City, UT United States; 2 Department of Information Systems WP Carey School of Business Arizona State University Phoenix, AZ United States; 3 Department of General Surgery and Community Medicine Research Center Keelung Chang Gung Memorial Hospital Keelung Taiwan; 4 Department of Chinese Medicine College of Medicine Chang Gung University, Taoyuan Chang Gung Taiwan

**Keywords:** data analytics, neural networks, phenotype, deep learning, electronic health records

## Abstract

**Background:**

Acute diseases present severe complications that develop rapidly, exhibit distinct phenotypes, and have profound effects on patient outcomes. Predictive analytics can enhance physicians’ care and management of patients with acute diseases by predicting crucial complication phenotypes for a timely diagnosis and treatment. However, effective phenotype predictions require several challenges to be overcome. First, patient data collected in the early stages of an acute disease (eg, clinical data and laboratory results) are less informative for predicting phenotypic outcomes. Second, patient data are temporal and heterogeneous; for example, patients receive laboratory tests at different time intervals and frequencies. Third, imbalanced distributions of patient outcomes create additional complexity for predicting complication phenotypes.

**Objective:**

To predict crucial complication phenotypes among patients with acute diseases, we propose a novel, deep learning–based method that uses recurrent neural network–based sequence embedding to represent disease progression while considering temporal heterogeneities in patient data. Our method incorporates a latent regulator to alleviate data insufficiency constraints by accounting for the underlying mechanisms that are not observed in patient data. The proposed method also includes cost-sensitive learning to address imbalanced outcome distributions in patient data for improved predictions.

**Methods:**

From a major health care organization in Taiwan, we obtained a sample of 10,354 electronic health records that pertained to 6545 patients with peritonitis. The proposed method projects these temporal, heterogeneous, and clinical data into a substantially reduced feature space and then incorporates a latent regulator (latent parameter matrix) to obviate data insufficiencies and account for variations in phenotypic expressions. Moreover, our method employs cost-sensitive learning to further increase the predictive performance.

**Results:**

We evaluated the efficacy of the proposed method for predicting two hepatic complication phenotypes in patients with peritonitis: acute hepatic encephalopathy and hepatorenal syndrome. The following three benchmark techniques were evaluated: temporal multiple measurement case-based reasoning (MMCBR), temporal short long-term memory (T-SLTM) networks, and time fusion convolutional neural network (CNN). For acute hepatic encephalopathy predictions, our method attained an area under the curve (AUC) value of 0.82, which outperforms temporal MMCBR by 64%, T-SLTM by 26%, and time fusion CNN by 26%. For hepatorenal syndrome predictions, our method achieved an AUC value of 0.64, which is 29% better than that of temporal MMCBR (0.54). Overall, the evaluation results show that the proposed method significantly outperforms all the benchmarks, as measured by recall, F-measure, and AUC while maintaining comparable precision values.

**Conclusions:**

The proposed method learns a short-term temporal representation from patient data to predict complication phenotypes and offers greater predictive utilities than prevalent data-driven techniques. This method is generalizable and can be applied to different acute disease (illness) scenarios that are characterized by insufficient patient clinical data availability, temporal heterogeneities, and imbalanced distributions of important patient outcomes.

## Introduction

### Background

Acute diseases and illnesses require timely and specialized care of patients whose conditions change rapidly, often within 48 hours of admission [1]. These diseases tend to evoke serious complications that develop quickly and can become fatal. Severe complications hinder patient recovery, substantially reduce their quality of life, create long-term impairments, and even cause death [2]. In general, a complication may have multiple subtypes or phenotypes, which signify and display distinct disease presentations [3,4]. Because phenotypes involve distinct symptoms and manifestations that require specific interventions, effective predictions of crucial complication phenotypes are crucial for physicians’ timely diagnoses and therapeutic treatments to improve patient management and reduce mortality rates.

Several data-driven techniques aim at identifying phenotypic expressions from electronic health records (EHRs) and use them to predict important clinical events, such as complications [5]. Predictive analytics helps advance such data-driven approaches to predict complication phenotypes; however, this ability presents various challenges in acute disease scenarios for several reasons. First, in the early stages of an acute disease, essential clinical features and characteristics (eg, risk factors) associated with complication phenotypes may not be sufficiently available to predict the phenotypic outcomes. This data insufficiency constraint can greatly reduce the predictive utilities of data-driven techniques [6]. Second, patients undergo various laboratory tests, medical examinations, and therapeutic treatments, which are administered at different frequencies and time intervals. The resulting temporal heterogeneities (eg, pattern, time interval, frequency) create additional difficulties for phenotype predictions. Third, for any particular acute disease, crucial complication phenotypes may arise in a relatively small proportion of patients, further causing imbalanced distributions of patient outcomes.

### Objectives

To address these challenges for effective complication phenotype predictions, we propose a novel recurrent neural network (RNN)-based method that incorporates a latent regulator (RNN-LR). Our method generates a temporal feature space representation with a recurrent neural network to cope with temporal heterogeneities in patients’ conditions and disease progression and then uses the latent regulator to mitigate the data insufficiency constraint. We used a data set of 6545 patients with peritonitis to evaluate the ability of the proposed method to predict acute hepatic encephalopathy (AHE) [7] and hepatorenal syndrome (HRS) [8]—two crucial phenotypes of hepatic complications that can develop after surgical procedures for peritonitis (eg, laparotomy). Although only a small proportion of patients with peritonitis develop these phenotypes, they are life threatening and difficult to predict [7,9].

The following three benchmark methods are evaluated in our study: temporal multiple measurements case-based reasoning (MMCBR) [10], time-aware long short-term memory (T-LSTM) network [11], and time fusion convolutional neural network (CNN) [12]. The results show that the proposed method significantly outperforms all the benchmarks, as measured by recall, F-measure, and area under the curve (AUC) while maintaining comparable precision values. Although our illustrative evaluation focuses on complication phenotypes of peritonitis, the proposed method is generalizable and applicable to predict phenotypes of other acute diseases that are characterized by insufficient patient clinical data availability, temporal heterogeneities, and imbalanced distributions of patient outcomes.

### Previous Work

Diseases can exhibit distinct phenotypic expressions [13]. For example, macrovascular disease spans six phenotypes, each associated with distinct anthropometric, clinical, and laboratory parameters [14]. Patients diagnosed with a particular disease can have complications pertinent to multiple phenotypes. Supported by accurate predictions of crucial complication phenotypes, physicians can improve their clinical decision making and patient management. To that end, predictive analytics empowers in-depth analyses of the rich patient clinical data in EHRs for the improved care and management of patients with acute diseases and illnesses [5,15].

#### Peritonitis

Peritonitis, an acute disease, is caused by the inflammation of the peritoneum [1] and often develops from bacterial or fungal infections [16]. Upon diagnosis with peritonitis, patients need immediate treatment (typically within 3 days), because it can progress rapidly and develop into life-threatening sepsis or septic shock [1]. Patients with peritonitis have higher mortality rates than those without peritonitis [17]. Several factors, such as age, sex, clinical conditions, and the living environment are associated with peritonitis-related mortality [17].

As two crucial phenotypic expressions of hepatic complications after peritonitis surgery, AHE and HRS can cause severe patient outcomes [7,18]. For patients with peritonitis who also have liver cirrhosis, intestinal bacterial overgrowth inside the body is responsible for hyperammonemia, which leads to AHE [19]. Similarly, HRS is a crucial complication phenotype of peritonitis with advanced cirrhosis too, that is characterized by renal failure and major disturbances in the circulatory function [8]. The underlying mechanisms of HRS may result from complex changes in splanchnic and general circulation, as well as systemic and renal vasoconstrictors and vasodilators [20]. Both phenotypes are clinically important; however, AHE is more severe than HRS because it can deteriorate in a matter of hours.

In general, AHE is diagnosed by liver specialists, whereas HRS is diagnosed by liver specialists and nephrologists. Clinically, the determination of each phenotype depends on laboratory results and the patient’s condition. Patients with AHE typically have hyperammonemia, hyperbilirubinemia, and central nervous system symptoms. Patients with HRS often display splanchnic arterial vasodilation and inflammation, which cause ascites and renal function impairment. Patients with AHE should receive lactulose and neomycin enema, which are infrequently used for other conditions according to the national health reimbursement policies in Taiwan. Thus, the occurrence of AHE can be assessed by the patient’s condition. Patients with HRS are usually prescribed albumin and terlipressin, which can then be used to determine the occurrence of HRS. Each phenotype has a particular ICD-9 code: 572.2 for AHE and 572.4 for HRS.

The heterogeneity and variability in manifestations of hepatic encephalopathy among patients make it difficult to assess or predict patient conditions [21,22]. Previous studies have shown that AHE may be present in 50%-70% of patients with peritonitis who have cirrhosis, including those with abnormalities detectable only with psychometric testing [23]. The clinical manifestations of AHE include brain dysfunction and deep coma [7]. This phenotype represents a vital disease entity because the risk of dying within a year exceeds 60% after its development [24]. Furthermore, patients with spontaneous bacterial peritonitis have an estimated 30% chance of developing HRS [20]. Clinically, the only curative treatment for AHE and HRS is liver transplantation, but systemic infection is a contraindication to liver transplantation. Without timely detection and proper interventions, AHE and HRS can develop rapidly, create patient impairments, lead to life-threatening conditions, and have alarming mortality rates [7,9]. These phenotypes have unique clinical characteristics and features that can be analyzed with data-driven analytics for prediction. Overall, existing data-driven techniques for phenotype predictions can be classified as rule-based, machine learning–based, or deep learning–based. We review the representative studies of each in the upcoming sections.

#### Rule-Based Phenotype Predictions

Rule-based techniques [25-29] use clinically important features to depict the underlying phenotypes. A typical rule-based technique iteratively updates heuristic rules until its sensitivity and specificity satisfy the prespecified thresholds. Developing heuristic rules is labor intensive and time consuming because it requires iterative rule generation and substantial involvement from human experts. The prediction of disease phenotypes entails the extraction of clinically important features; essential features and their combinations in turn indicate the underlying disease phenotype. Guided by domain knowledge, previous research has developed heuristic rules to extract essential features (eg, medications, laboratory results, diagnoses) from EHRs for phenotype predictions, and then updated the extracted rules iteratively until sensitivity and specificity reached the prespecified levels. For example, the rule-based eMERGE technique uses EHRs, in combination with DNA biorepositories, to identify diabetic phenotypes and medication-induced liver lesions [29].

#### Machine Learning–Based Phenotype Predictions

Machine learning techniques coupled with EHRs can support and enhance the care and management of patients with peritonitis. For example, by integrating cellular and soluble biomarkers, support vector machines and tree-based algorithms can help physicians in predicting pathogen-specific immune responses of patients with peritonitis and guide them to formulate optimal antibiotic and operative therapies [30]. Previous research has also applied machine learning algorithms to predict phenotypes [5,28].

Existing machine learning–based techniques can be categorized as clustering analysis, graph-based learning, and probabilistic modeling. Techniques that rely on clustering analysis create phenotype clusters, such that patients in the same phenotypic cluster are more similar to one another than to patients in a different cluster. In essence, clustering analysis–based techniques [5,31] generate patient clusters so that patients with similar phenotypic expressions are in the same cluster. They usually use cross-sectional patient data to produce distinct clusters at a given time or analyze longitudinal clinical data to infer phenotypes that remain consistent over time [5]. However, existing clustering algorithms cannot deal robustly with high-dimensional patient data, and their applications are restricted to smaller, more homogenous data sets [5]. Most clustering-based techniques are applied to patient data at a single time point. Thus, clustering analyses of temporal data would require multiple applications of the chosen technique at different time points [31], further creating instability in the resulting phenotype clusters.

Graph-based techniques [32-34] can cope with temporal heterogeneities in longitudinal patient data (eg, pattern, time interval, frequency). They often assume sequential linkages of distinct clinical events and represent those events as temporally connected nodes in a graph [32]. However, this assumption does not always hold clinically. For example, patients frequently and concurrently receive multiple laboratory tests, treatments, or therapeutic procedures. Moreover, the graph construction process does not include laboratory results (values) that can be essential for inferring clinical outcomes [32]. In addition, probabilistic modeling can uncover the underlying phenotypes. For example, Pivovarov et al [35] propose UPhenome—an unsupervised, generative probabilistic model that can learn phenotypes from heterogeneous patient data. To identify chronic obstructive pulmonary disease subtypes that are similar in progression characteristics, Ross et al [36] develop a novel Bayesian nonparametric model that uses disease trajectory to represent the underlying biological or genetic similarity within the subtype.

The crucial peritonitis complication phenotypes that we study—AHE and HRS—can occur rapidly without any predictive signs, thereby hindering the use of conventional machine learning techniques for predictions. Recent advancements in deep learning promise better predictions of patient outcomes [37,38] because they can learn from clinical sequences to account for complex patterns and relationships in sequential inputs. To illustrate, representation learning can extract complex relationships and nonlinearities among temporal events. Moreover, deep learning architectures, such as recurrent and convolutional neural networks, can be applied to better predict patient outcomes [39-41]. In the following sections, we review representative deep learning–based techniques that can deal with high-dimensional and temporally heterogeneous patient data.

#### Deep Learning–Based Phenotype Predictions

The use of predictive analytics for clinical decision support and patient management often involves large amounts of heterogeneous patient clinical data and needs to consider temporal relationships [42]. Fueled by fast-growing computational power and proliferating EHRs, deep learning has been applied in a broad array of diagnostic tasks, including those related to phenotypes [43,44]. For example, reconstructed RNNs with rectified linear units can impute missing values in genotype data to predict phenotype sequences [45]. Deep autoencoder techniques for unsupervised feature learning help clinicians in identifying acute leukemia phenotypes [46]. By combining latent representation learning of deep neural networks and causal inferences, Kale et al [40] discovered latent phenotypes that are causally predictive of clinical outcomes in patients in the intensive care unit. Moreover, deep RNNs can model multivariate clinical time series in a large data set and then transfer the knowledge to the limited labeled instances to classify the phenotypes of patients in the intensive care unit [47]. Existing literature suggests the value and feasibility of using deep learning in different diagnostic tasks and clinical contexts.

Particularly, EHRs contain rich, longitudinal patient clinical data that can be modeled as RNNs that can represent patients’ records in an accurate and robust way [48]. These networks are effective for modeling patient (clinical) records as temporal logs of diagnostic results. For a particular patient, the state of disease or illness at time *t* is a summary of the diagnostic records before *t.* With each record represented as a feature vector, the vectors at different time points can provide sequential inputs to an RNN. The outputs at time *t*+1 can be used to produce a vector that represents the patient’s state at *t+*1*.* Such patient-level vector representations can be further input into other (hidden) layers of the neural network to predict clinical outcomes (eg, readmission, mortality, complications). For predictive analytics in clinical scenarios, RNN-based deep learning architectures may be advantageous over traditional machine learning techniques. For example, an RNN can reduce or prevent adverse drug events by integrating heterogeneous, multidimensional drug data from different sources [49]. In addition, by coping with various clinical and temporal data, an uncertainty-aware convolutional RNN can predict patient mortality, with uncertainty denoting the irregular time intervals in patients [50].

#### Cost-Sensitive Learning

Many clinical diagnoses feature relatively few crucial cases among patients, which need to be properly addressed by data-driven techniques for prediction. If a sample has a substantially fewer number of minority class cases, standard classifiers generally cannot perform well because their predictions tend to steer toward the majority class. Cost-sensitive learning can address the imbalanced distributions of patient outcomes in a sample. It considers the misclassification cost (and possibly other costs) by assigning a high penalty (cost) to the misclassifications of a minority-class instance, without modifying the original data distribution in the sample [51]. Such learning essentially shifts the bias of a classification model in the favor of the minority class. By adjusting the costs associated with different misclassified labels [52], and with the goal of minimizing the total cost, cost-sensitive learning can produce greater predictive utilities. In many clinical scenarios, the minority class is relatively more important and has a higher misclassification cost. However, the overall performance of a classification model, whether machine learning– or deep learning–based, can be dominated by the majority-class instances. This issue may be addressed by combining evaluation results (eg, F-measure, AUC) and the costs associated with different outcome classes (eg, complication phenotypes) to optimize the cost parameter for effective classifications [51].

#### Research Gaps

This review of extant literature reveals several gaps. First, existing prediction techniques may be inadequate or ineffective for acute disease scenarios because previous phenotype research focuses largely on patients with chronic diseases [53,54], whose clinical conditions change less drastically than those of patients with acute diseases. In addition, patients with chronic diseases usually have fewer complications that develop rapidly and have more clinical data available for predictions compared with patients with acute diseases. Second, most previous research works [5,40,46,55] tend to overlook the data insufficiency constraint, which limits the use of early disease stage patient data to build effective computational models for predicting complication phenotypes. Several studies have identified disease phenotypes by assuming full patient data availability [40,46]; however, clinical data captured in the early stages of an acute disease may lack essential information for predicting complication phenotypes. Some important clinical characteristics and factors of complication phenotypes may be available in the early stages but are not sufficiently informative for predicting phenotypic outcomes. Third, complication phenotypes associated with an acute disease often have an imbalanced distribution of different outcomes. Fourth, the clinical efficacy of data-driven techniques for complication phenotype predictions still requires adequate empirical evaluations, especially in acute disease situations that feature data insufficiencies and imbalanced distributions of patient outcomes.

Effective complication phenotype predictions need to address these challenges and consider patients’ heterogeneities and disease progression variations over time while coping with the data sufficiency constraint. We propose a deep learning–based method that leverages temporal feature space representation to address temporal heterogeneities in patient data. Although previous research works have acknowledged the importance of unobserved latent factors for influencing phenotypes [40,56], few studies have explicitly considered such factors for phenotype predictions. As a remedy, we incorporate a latent parameter matrix to account for unobserved (subsequent) patient condition and disease progression variations. In addition, our method addresses missing values in patient data and includes cost-sensitive learning, which can address imbalanced outcome distributions by combining evaluation results (F-measure and AUC) and the cost associated with each complication phenotype to optimize cost parameters for an improved predictive performance.

## Methods

**Figure 1:**
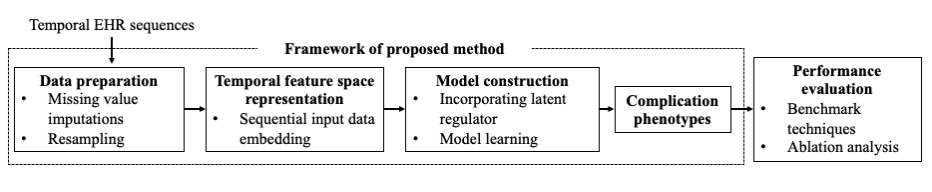
Overall processing of recurrent neural network-latent regulator. EHR: electronic health record.

We elaborate on the proposed method in Figure 1. As shown in the figure, this method involves data preparation, temporal feature space representation, model construction, and model evaluation.

### Data Preparation

Missing data prevail in many clinical scenarios and create fundamental challenges for predictive analytics [57]. Patients with acute diseases are often closely monitored with various laboratory tests, but missing values arise when the test results are not properly and consistently recorded because of the physician’s preference, recording errors, or other reasons. For data preparation, we perform expert-guided feature selection to identify the clinical attributes and laboratory tests that are essential to a severe complication and then employ a deep autoencoder-based model to impute missing values for these features. The deep autoencoder model [58] identifies patients similar to the focal patient and uses their attribute values to infer and replace the patient’s missing values [59]. Because only a relatively small proportion of patients may develop severe complications, we apply the SMOTEENN (Synthetic Minority Oversampling Technique–Edited Nearest Neighbors) algorithm [60] to address the imbalanced distributions of different outcome classes.

### Temporal Feature Space Representation

Patient data, including vital signs and laboratory results, are longitudinal and pertain to different clinical events over time. A clinical sequence reflects the patient’s disease progression and has heterogeneous characteristics that may prevent clinically actionable insights. To extract acute disease progression from sequential (clinical) events, we apply sequence embedding, which is a feature leaning technique that projects sequential events into vectors of numeric numbers. In general, patients sharing similar clinical conditions are closer in distance than otherwise. Therefore, we used a temporal representation to depict each patient’s disease progression. We assume that a patient *p* has a set of temporal clinical events 
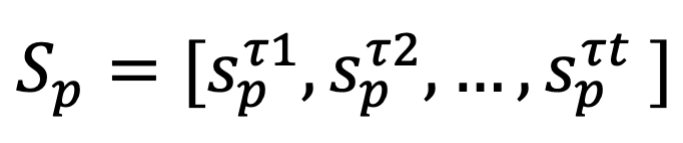
 that occur between time 
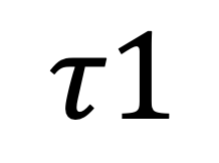
 and 
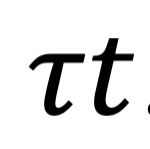
 . At each time point
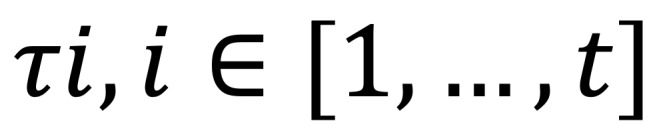
, a patient may have multiple temporal features (eg, test results, diagnoses), denoted by 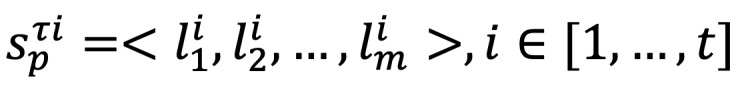 , where *m* indicates the number of diagnosis categories at 
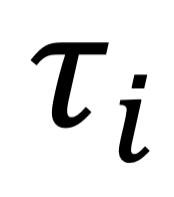
 . In addition, each patient has demographic data (eg, sex, age), represented as 
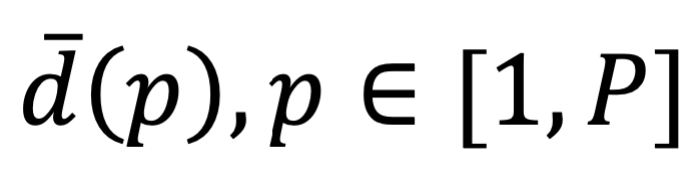
 , where *P* is the total number of patients. With observed clinical (event) sequences, we can construct a temporal feature representation (
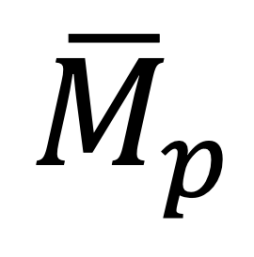
 ) for that patient:

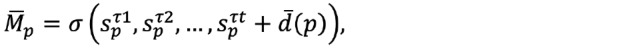



Where 
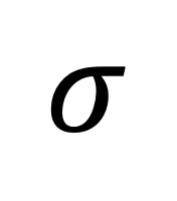
 is a function that can project the temporal clinical sequences to the temporal feature representation (
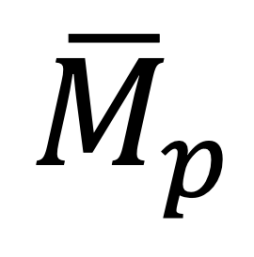
).

### Model Construction

Variations that exist in patients’ conditions and disease progression cannot be fully explained by patients’ demographics, laboratory results, and therapeutic (surgical) data [61]. Therefore, we include an additional parameter matrix 
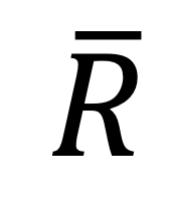
, which serves as a latent regulator to account for disease progression information or underlying mechanisms related to complication phenotypes. In addition, 
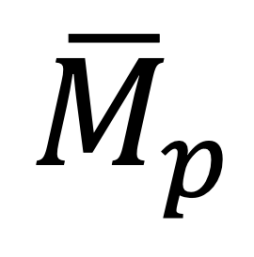
 refers to the disease progression space and comprises information extracted from clinical data available in early disease stages; that is, 
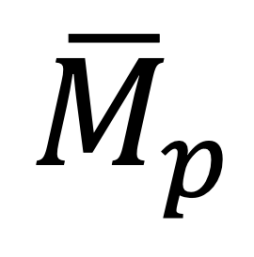
 is the temporal feature space extracted from patients’ clinical data. The data available in the early stages of an acute disease are usually limited and cannot reveal a patient’s subsequent progression or effectively predict complication phenotypes. To alleviate this constraint, 
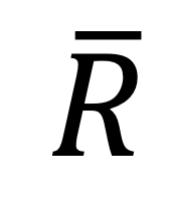
 acts as a latent regulator, independent of the disease progression space (
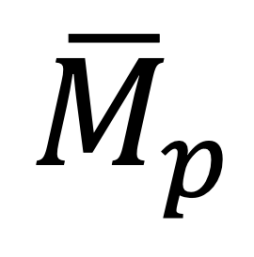
) to account for unobserved variations in the subsequent patient condition and disease progression.

We assume a combined effect of 
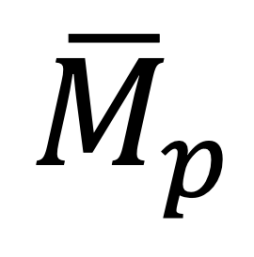
 and 
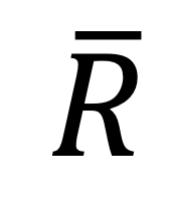
, for which 
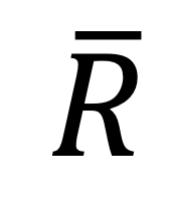
 is generalized with iterative clinical feature updates, according to



where γ is the learning rate, and 
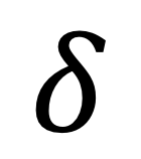
 indicates the minimum number of iterations required to converge 
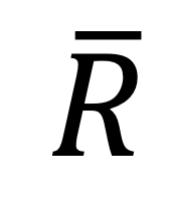
. During model learning, 
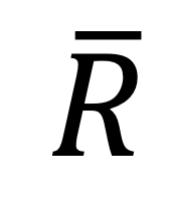
 gradually converges to a stable range, as depicted in 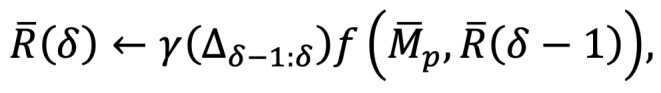. For testing, the parameter matrix (
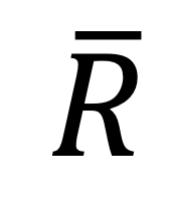
) facilitates phenotypic predictions for individual patients.

**Figure 2:**
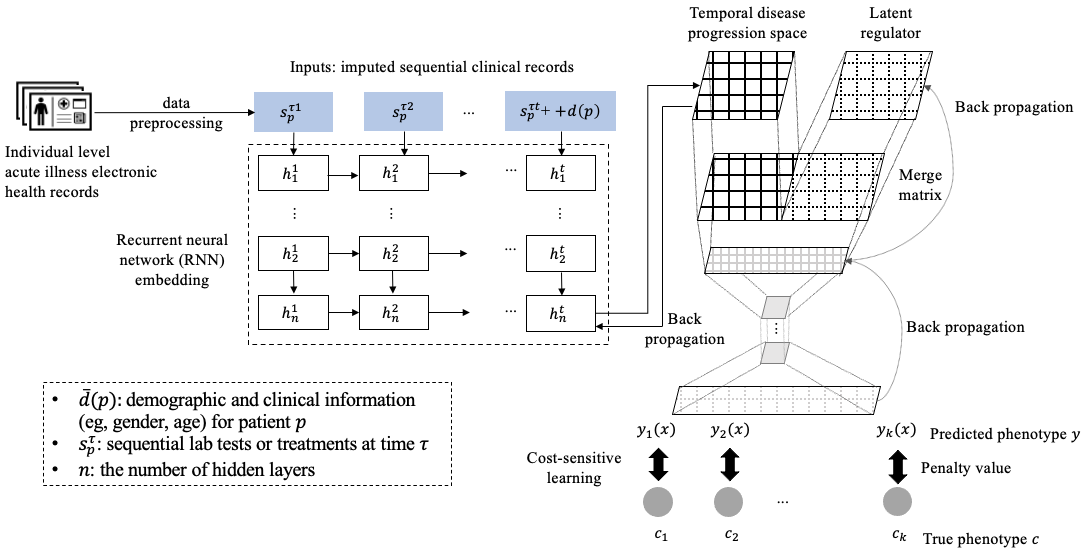
Proposed latent regulator-embedded recurrent neural network method.

Although severe complications represent the minority class in patient data, they have profound effects on patient outcomes and health care costs. Hence, we employ cost-sensitive learning to better predict the minority class, according to the respective misclassification costs, by applying the cost matrix to penalize incorrect predictions (misclassifications). Figure 2 presents the proposed RNN-LR method.

Our method minimizes the expected costs of incorrect phenotype predictions, calculated as:

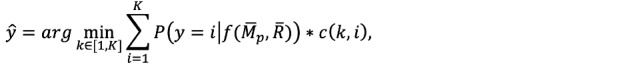



where 
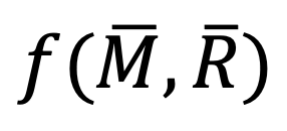
 is the learning function of 
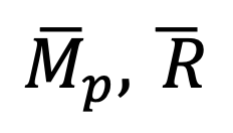
 and reveals the combined effect of disease progression and a latent regulator on prediction, *K* denotes the total number of classes, *c*(*k, i*) indicates the cost of misclassifying an instance of class *k* as class *i*. 
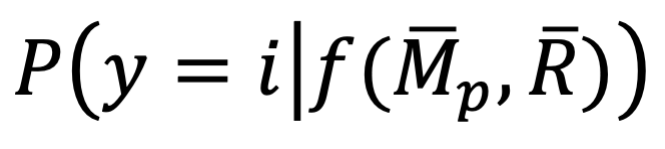
 estimates the probability of class *i*, given (
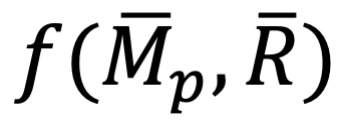
). As shown in Figure 2, 
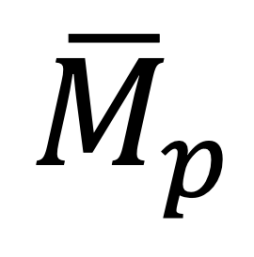
 and 
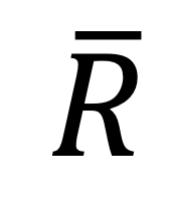
 serve as input to the second multilayer neural network and thereby are mapped into the phenotype space. We use a SoftMax function to estimate the probability that an instance is classified as each distinct outcome class. In the output layer, the *i*th node contains weight (
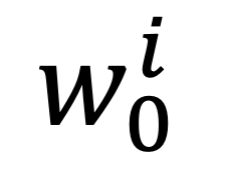
) and bias (
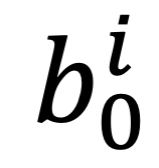
). For each phenotype outcome class, the probability of phenotype *i,* given 
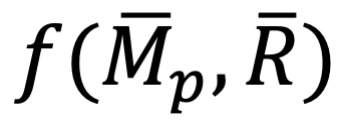
 can be calculated as:

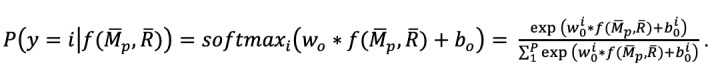



Finally, we employ cross-entropy as a loss function to learn and optimize the model parameters:

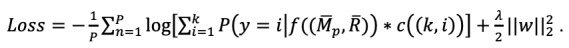



Thus, parameters get updated through Adam optimizer and back propagation.

### Data

#### Data Sources

We obtained a clinical data set from the Department of Laboratory Medicine at the Chang Gung Memorial Hospital, which is accredited by the Taiwan Accreditation Foundation and approved by the American College of Pathologists [62,63]. This data set consists of 10,354 records pertaining to 6545 patients who underwent peritonitis surgery between 2003 and 2015. Designated professionals at the hospital integrated patient records in EHRs, according to the common format of the Chang Gung Research Database (CGRD) that provides standardization and facilitates data extraction, transformation, and loading for analyses [64].

The CGRD links directly to the National Health Insurance Research Database (NHIRD), which informs reimbursement decisions [62,63]. To prevent fraud and contain costs, the National Health Insurance Administration of the Ministry of Health and Welfare performs frequent, random audits. Thus, the data in the NHIRD and CGRD are reliable and accurate. Information systems professionals at the hospital also assessed the data conversion process and integrity to ensure that all patient records (including diagnoses and laboratory results) are correctly transferred to the CGRD without errors or losses [64]. Experienced personnel from the Research Institute of Chang Gung Memorial Hospital assisted us in compiling the data set. In particular, they performed data preprocessing and consolidation to ensure that each patient’s records were collected within the same time interval.

#### Data Processing and Details

In the data set, each laboratory test has a time stamp that indicates when the results are available (reported). We used the date of peritonitis surgery as the starting point and collected patient data over the next 3 days. This procedure ensured that all patient records were collected within the same time interval after surgery. Each patient had at least one clinical record within the three-day window. Specifically, 3665 patients had 1 clinical record, 1951 patients had 2, and 929 patients had 3 clinical records. Each record contains the patient’s demographic and clinical data (eg, age, sex, comorbidity) and potentially multiple laboratory results, which we used to predict the complication phenotypes (AHE, HRS, or neither).

Table 1 summarizes the patient demographic and clinical variables in the data set. We adopted the International Classification of Diseases, Ninth Revision, Clinical Modification comorbidity coding algorithm [65] to define the Charlson comorbidities in administrative and clinical data. Accordingly, the comorbidity types in our study span 17 categories, from mild liver disease to moderate or severe liver disease to myocardial infarction. Among the 6545 peritonitis patients in the data set, 41 developed AHE, and 174 developed HRS, that is, the distribution of different outcome classes (AHE, HRS, or neither) is highly imbalanced. Furthermore, AHE and HRS have profound implications for patient outcomes and mortality. For example, 9 of the 41 patients with AHE in the sample died, with a mortality rate of 22%; patients with AHE or HRS had an average length of stay of 25 days (hospitalization), whereas patients without these phenotypes had an average of 17 days.

**Table 1:** Summary of patient demographic and clinical variables in the peritonitis data set.

Variables		Type	Value or range	Description
**Variable name**				
	Patient_ID	String	Unique patient ID.	Patient ID
	Inpatient_ID	String	Unique inpatient ID	Inpatient ID
	Sex	Integer	0 or 1	Male or female
	Age (<20, 20-60, or >60 years)	Integer	(0-102)	Patient’s age
**Inpatient variables**				
	DGTM^a^	Integer	(0-136)	The number of hospitalizations before peritonitis surgery
	Comorbidities (including malignant tumor)	String	[Mild liver disease, …, Renal disease]	17 classified comorbidities that include liver disease, according to ICD-9^b^ coding algorithms [65]
	Operation category	String	0, 1, 2, 3, 4, 5, or 6	Seven categories in total (adopted from ICD-9-CM^c^): cholecystitis or cholangitis, appendicitis, hollow organ perforation, bowel ischemia, intestinal obstruction, hernia with bowel gangrene, hernia with bowel obstruction
	Complication pneumonia	Integer	0 or 1	Whether a patient has pneumonia
	Complication UTI^d^	Integer	0 or 1	Whether a patient has UTI
	Complication SSI^e^	Integer	0 or 1	Whether a patient has SSI
**Laboratory test results**				
	Albumin	Float	(0.19-5.5g per dL)	Serum albumin level
	Amylase	Float	(5-11873 U per L)	Serum amylase
	BNP^f^	Float	(21.49-4942 pg per ml)	Levels of B-type natriuretic peptide
	BUN^g^	Float	(1-281 mmol per L)	Blood urea nitrogen
	Band^h^	Float	(0.3-76)	Band neutrophil
	Ca^i^	Float	(0.8-14.49 mg per dL)	Calcium in blood
	CR^j^	Float	(0.07-34.2 mg per dL)	Serum creatinine
	CRP^k^	Float	(0.2-679.2 mg per L)	C-reactive protein test
	Hematocrit	Float	(1-63.2%)	Hematocrit test (proportion of red blood cells in the blood)
	INR^l^	Float	(0.79-12)	International normalized ratio of prothrombin time
	K^m^	Float	(1.60-18.86 mEq per L)	Blood potassium test
	Lactate	Float	(5.49-240.6 mmol per L)	Level of lactic acid
	Leukocyte (WBC^n^)	Float	(0.2-141.5×10^9^ per L)	White blood cell count
	Platelets	Float	(0.7-1373×10^9^ per L)	The number of platelets in blood
	Prealbumin	Float	(2-44.59 mg per dL)	Prealbumin in blood
	Procalcitonin	Float	(4.56-45.49 µg per L)	Blood test to diagnose sepsis
	Total bilirubin	Float	(0.1-56.54 mg per dL)	Total amount of bilirubin in blood
	Na^o^	Float	(10-190 mEq per L)	Blood sodium test
**Target variable (crucial complication phenotype to be predicted)**				
	Complication phenotype	Categorical	AHE^p^, HRS^q^, or Neither	

^a^DGTM: number of hospitalization before peritonitis surgery.

^b^ICD-9: International Classification of Diseases, Ninth Revision.

^c^ICD-9-CM: International Classification of Diseases, Ninth Revision, Clinical Modification.

^d^UTI: urinary tract infection.

^e^SSI: surgical site infection.

^f^BNP: B-type natriuretic peptide.

^g^BUN: blood urea nitrogen.

^h^Band: bandemia.

^i^Ca: calcium.

^j^CR: creatinine.

^k^CRP: C-reactive protein.

^l^INR: international normalized ratio.

^m^K: potassium.

^n^WBC: white blood cell.

^o^Na: sodium.

^p^AHE: acute hepatic encephalopathy.

^q^HRS: hepatorenal syndrome.

For the 41 patients with AHE in the sample, the complication phenotype occurred between 6 and 17 days after peritonitis surgery, with a mean of 13 days. For the 147 patients with HRS, the complication phenotype developed between 4 and 47 days after the surgery, with a mean of 14 days. In their clinical trial, Huang et al [66] reported a survival curve that indicated that most patients discontinued antibiotic treatment within 3 days after peritonitis surgery, suggesting that AHE and HRS seldom occur within those 3 days. In general, complication phenotypes, including AHE and HRS, arise approximately 2 or 3 weeks after peritonitis surgery [66,67]. For example, HRS is characterized by a rapid, progressive impairment of renal function. Furthermore, it develops, on average, about 2-3 weeks after peritonitis surgery [68,69]. Similarly, patients develop AHE approximately 2 weeks after peritonitis surgery [70,71]. The combined evidence from the relevant literature and clinical findings indicates the appropriateness of using the data available 3 days after surgery to predict subsequent AHE and HRS occurrences.

### Descriptive Statistics

We provide some descriptive statistics related to gender, the number of hospitalizations before peritonitis surgery, and different complication phenotypes (ie, AHE, HRS, and neither) in the data set. The average number of hospitalizations before surgery was slightly lower for AHE and HRS than for neither: 2.6 for AHE, 2.4 for HRS, and 2.7 for neither. Both male and female patients had a similar number of hospitalizations: approximately 2.5 times. Female patients with neither of the diseases had more hospitalizations (about 3.2 times) than their male counterparts. These differences in part reflect the risk: both AHE and HRS may arise abruptly, even without many previous hospitalizations.

**Figure 3:**
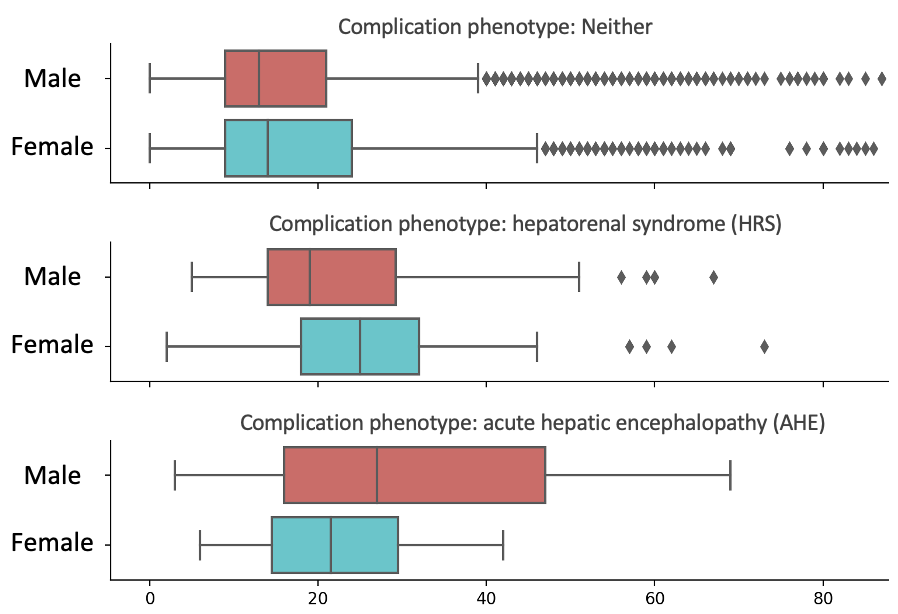
Analysis of sex, complication phenotype, and the length of stay in the data set.

We also analyzed the relationships of sex, complication phenotypes, and the length of stay after surgery. As shown in Figure 3, both AHE and HRS induce longer lengths (20 days+) after surgery, whereas for neither, the length of stay was approximately 17 days. The longer length of stay associated with AHE and HRS again underscores the importance of phenotype predictions. For AHE, male patients had a longer length of stay than female patients, but we observed an opposite pattern for HRS.

### Evaluation Design

#### Benchmark Techniques

In total, three prevalent techniques were included in the evaluation as benchmarks: temporal MMCBR [10], time fusion CNN [12], and T-LSTM [11]. First, temporal MMCBR performs clustering analyses to identify similar (patient) temporal sequences in a sample [10]; therefore, it can handle temporal patient data that vary in their time intervals and granularity. Second, time fusion CNN, a deep learning–based technique, learns patient representations and measures pairwise similarity in temporal patient data to capture important characteristics specific to individual patients [12]. Third, patient subtyping through T-LSTM, another deep learning–based technique, can cope with patient data that feature temporal heterogeneities by employing autoencoders to learn patient representations, which helps cluster patients into subtypes [11]. Our benchmarks do not include graph-based techniques because concurrent laboratory tests in the data set make them inadequate for representing patient conditions and disease progression in a 2D graph. We also exclude probabilistic modeling that offers limited predictive utilities in situations involving imbalanced samples. In the evaluation, the proposed method and all benchmark techniques employed the same cost-sensitive matrix.

#### Implementation and Parameter Tuning

All the evaluations were performed on a computer with a dual-core processor of 2.7 GHz and 8 GB of memory, running macOS Catalina. We used the SMOTEENN algorithm from the Python imbalanced-learn library and applied the Python Shapley Addictive Explanations (SHAP) package to obtain SHAP values for the feature importance analysis. The proposed method and benchmark techniques were implemented using PyTorch. Our method constructs an 8-layer RNN to map the disease progression space with a latent regulator and adopts a multilayer perceptron neural network with three dense layers to predict complication phenotypes. Specifically, the RNN embedding produces a 2D vector, 8×8 in size, which depicts the temporal disease progression space. We randomly split the data set into 80% for training and 20% for testing. The testing set had 12 AHE cases, 35 HRS cases, and 1262 neither cases. For misclassified labels, we set the initial cost parameter for each phenotype (AHE or HRS) to 200, in line with a related research [72]. We performed a series of parameter tuning analyses, and then used the results to determine essential hyperparameter values (Table 2), including an optimal number of layers for each neural network.

**Table 2:** Essential hyperparameters used in the proposed method.

Parameter	Value
Learning rate (γ)	0.01
Latent regulator size	8×8
Drop rate	0.2
Number of layers	8
Units in each layer	16
Weight decay (λ)	0.005

**Figure 4:**
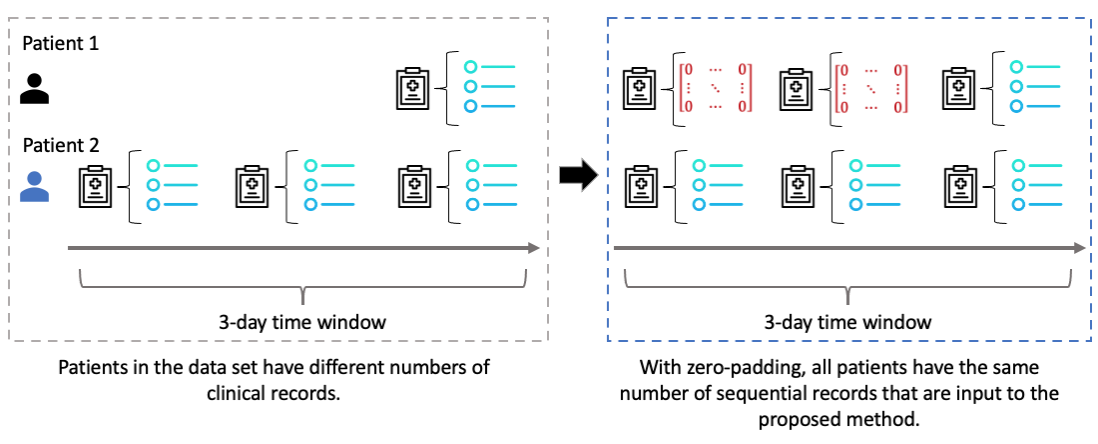
Use of zero padding to prepare clinical records for the proposed recurrent neural network-latent regulator method.

A clinical record contains the results of the laboratory tests prescribed by the physician, that is, a record has one timestamp. As noted, patients in the data set have different numbers of records within the 3-day window after peritonitis surgery, which are used for model construction. Because the proposed RNN-LR method requires the same number of clinical records for each patient, we employed zero padding to ensure that each patient had three sequential records. As a result, the data set contains a total of 19,635 clinical records: 6545 patients × 3 (sequential) records. We illustrate the zero-padding process in Figure 4. If a patient has only one clinical record within the 3-day window, we place that record at the end of the sequence and fill the first 2 records with zeroes, according to the length of the longest sequence (three). Hence, our method uses input sequences of the same length for model training. 

**Figure 5:**
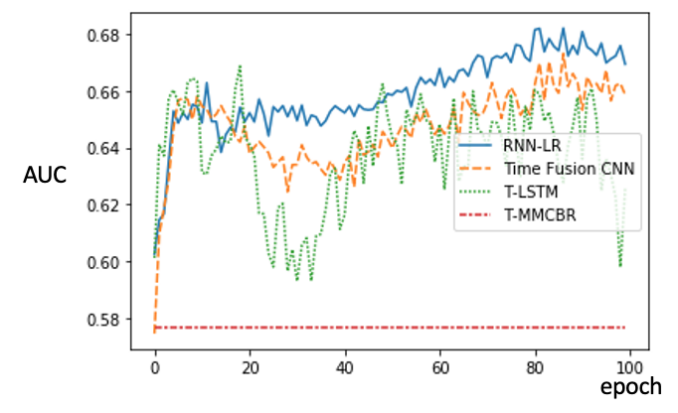
Analysis of learning curves of the proposed method versus benchmark techniques. AUC: area under the curve; CNN: convolutional neural network; MMCBR: multiple measurement case-based reasoning; RNN-LR: recurrent neural network-latent regulator; T-LSTM: time aware long short-term memory.

We used the ReLU for activation and the cross-entropy function for optimization. Finally, the Adam optimizer was applied to update the model parameters. Figure 5 presents the learning curves of the proposed method versus benchmark techniques [73]. As shown, the hyperparameters appear to converge toward optimality after 100 epochs. Notably, our method consistently achieves a greater AUC than the benchmark techniques after 60 epochs.

#### Performance Measures

We evaluated predictive performance in terms of recall, precision, F-measure, and AUC. In line with previous research works [74,75], we adopted the one-against-all strategy to examine the respective techniques across different performance measures and outcome classes. To illustrate, we combine HRS and neither as a single class to calculate the precision, recall, and F-measure while evaluating AHE predictions. This approach reduces 3 outcome classes to 2 (AHE and no AHE), with AHE as the positive class and no AHE as the negative class. Similarly, we consider HRS as the positive class and no HRS as the negative class while assessing HRS predictions. Recall, or sensitivity, indicates the fraction of correctly predicted positive observations among all actual positive-class observations, calculated as:

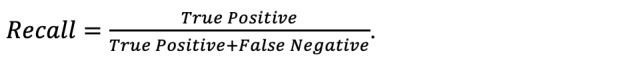



Precision, or positive predictive value, denotes the ratio between the correctly predicted positive (or negative) observations and the total predicted positive (or negative) observations, calculated as:

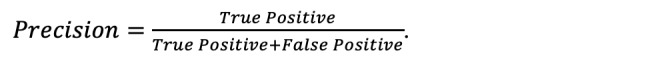



A high recall value reflects the ability to predict patients who will develop AHE or HRS, whereas a high precision value signals a low false positive rate. The F-measure is the harmonic mean of precision and recall, with 1 indicating the best performance and 0 indicating the worst [76], calculated as:

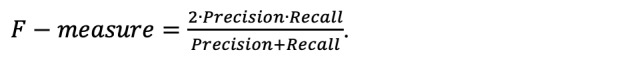



Finally, the AUC depicts a technique’s overall ability to distinguish different outcome classes across various threshold values. Because we apply the one-against-all strategy in the evaluation, the AUC reveals a technique’s performance relative to a random classification, without any biases associated with the sample size used in the evaluation.

## Results

### Imputation Performance

We evaluated the performance of several prevalent missing value imputation techniques: multivariate imputation by chained equations [77], SoftImput [78], a K-nearest neighbors technique [79], and a deep autoencoder model [58]. To compare their effectiveness, we randomly removed 20% and 30% of the laboratory results from the data set, applied each technique to impute the missing values, and then calculated the normalized root mean squared error between the predicted and holdout values. The NRMSE is the difference between the imputed and the holdout values divided by the average value of the complete data. As shown in Table 3, the deep autoencoder model, which we incorporated into the proposed method, consistently exhibits the best imputation performance consistently.

**Table 3:** Missing value imputation performance of respective techniques.

Technique (reference)	NRMSE^a^	
	20% imputation performance	30% imputation performance
MICE^b^ [77]	0.8479	0.8886
SoftImpute [78]	0.8546	0.9044
KNN^c^-based imputation [79]	1.0209	1.0044
Deep autoencoder model [58]	0.7926	0.8453

^a^NRMSE: normalized root mean squared error.

^b^MICE: multivariate imputation by chained equations.

^c^KNN: K-nearest neighbors.

### Prediction Performance Evaluation

Table 4 presents the results of prediction evaluation. Overall, the proposed method outperforms all the benchmarks for predicting AHE and HRS, as measured by recall, F-measure, and AUC. Because recall indicates the ability to identify patients who are likely to develop AHE or HRS, it is arguably more important than precision. For predicting AHE, our method achieves 27%-147% improvements in recall, 26%-64% in AUC, and 56%-100% in the F-measure compared with the benchmarks. For HRS predictions, we observed 5%-300% improvements in recall, up to 19% improvement in AUC, and up to 30% in the F-measure. The recall level achieved by our method, recorded at 0.42 for AHE and 0.40 for HRS, is significantly higher than that of the best-performing benchmark (T-LSTM). Similarly, the AUC values attained by the proposed method, 0.82 for AHE and 0.64 for HRS, are significantly greater than those of T-LSTM or time fusion CNN. Jointly, the F-measure and AUC values attained by the proposed method indicate its greater effectiveness in predicting the crucial complication phenotypes than the benchmark techniques because of its high recall and comparable precision. Together, these results reveal that the proposed method can help physicians concentrate on patients who are more likely to develop severe complications.

**Table 4:** Prediction performance of the proposed method versus benchmark techniques.

Technique and outcome class		Precision	Recall	F-measure	AUC^a^
**Temporal MMCBR^b^**					
	AHE^c^	0.20	0.17	0.18	0.50
	HRS^d^	0.09	0.10	0.10	0.54
	Neither	0.97	0.96	0.96	0.54
**Time fusion CNN^e^**					
	AHE	0.12	0.33	0.18	0.65
	HRS	0.06	0.38	0.10	0.67
	Neither	0.97	0.81	0.88	0.63
**T-LSTM^f^**					
	AHE	0.09	0.33	0.14	0.65
	HRS	0.05	0.27	0.09	0.68
	Neither	0.97	0.84	0.90	0.63
**RNN-LR^g^**					
	AHE	0.21	0.42	0.28	0.82
	HRS	0.08	0.40	0.13	0.64
	Neither	0.98	0.85	0.91	0.66

^a^AUC: area under the curve.

^b^MMCBR: multiple measurement case-based reasoning.

^c^AHE: acute hepatic encephalopathy.

^d^HRS: hepatorenal syndrome.

^e^CNN: convolutional neural network.

^f^T-LSTM: time aware long short-term memory.

^g^RNN-LR: recurrent neural network-latent regulator.

**Figure 6:**
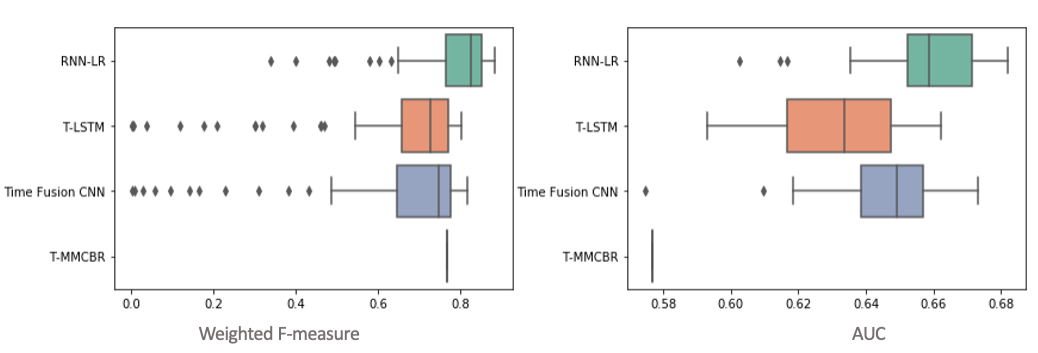
Predictive performances of our method versus benchmarks, 100 evaluation trials. AUC: area under the curve; CNN: convolutional neural network; MMCBR: multiple measurement case-based reasoning; RNN-LR: recurrent neural network-latent regulator; T-LSTM: time aware long short-term memory.

We performed paired *t* tests to examine whether the performance improvements achieved by our method over benchmark techniques are significant. Specifically, we independently evaluated each technique 100 times, and then used the results to test the significance of the performance differentials. As shown in Figure 6, the improvements in the weighted F-measure and AUC associated with our proposed method are statistically significant at *P*<.05. Table 5 details the paired *t* test results.

**Table 5:** Paired *t* test results of improvements by our method over benchmark techniques.

Proposed method (RNN-LR^a^)	AUC^b^			Weighted F-measure				
	T-LSTM^c^	Time fusion CNN^d^	Temporal MMCBR^e^		T-LSTM	Time fusion CNN	Temporal MMCBR	
*P* value	*<*.001	<.001	<.001		*<*.001	<.001	<.05	

^a^RNN-LR: recurrent neural network-latent regulator.

^b^AUC: area under the curve.

^c^T-LSTM: time aware long short-term memory.

^d^CNN: convolutional neural network.

^e^MMCBR: multiple measurement case-based reasoning.

Table 6 presents the confusion matrix created by testing the case predictions with our method. Because neither account for the majority of the peritonitis sample, we observe a tendency to predict AHE or HRS as neither.

**Table 6:** Confusion matrix of test sample predictions by the proposed method.

Actual outcome class	Predicted outcome class		
	Predicted neither	Predicted AHE^a^	Predicted HRS^b^
Actual neither	1079	18	165
Actual AHE	7	5	0
Actual HRS	20	1	14

^a^AHE: acute hepatic encephalopathy.

^b^HRS: hepatorenal syndrome.

**Figure 7:**
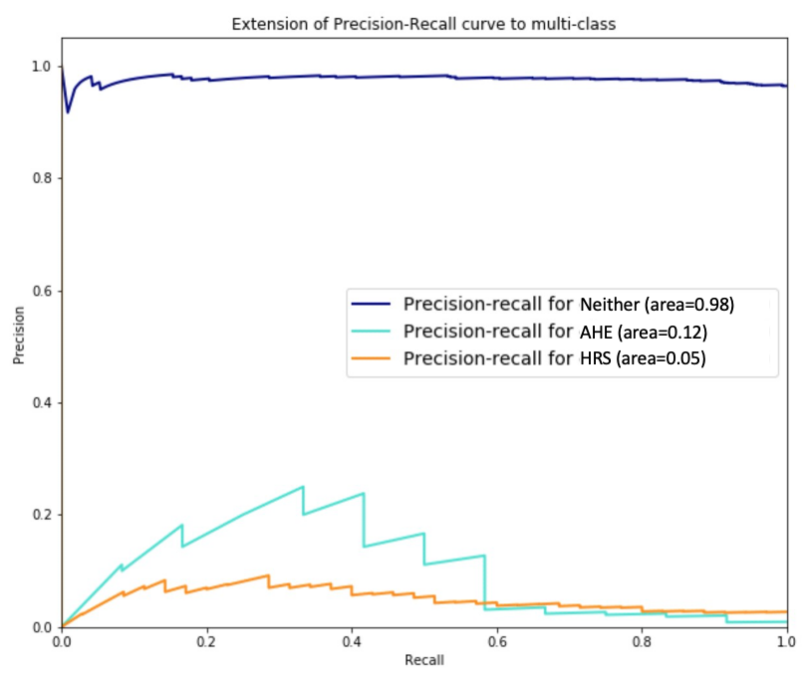
Precision-recall curves for the proposed method regarding acute hepatic encephalopathy, hepatorenal syndrome, and neither of these conditions.

The relatively low precision values of both our proposed method and the benchmark techniques can be attributed to the highly imbalanced outcome class distributions: AHE and HRS cases only account for 0.6% and 1.1% of the sample, respectively. Figure 7 depicts the precision-recall curves that reveal their trade-off across different thresholds. Although both AHE and HRS have low precision and recall values because of their imbalanced distributions in the sample, a higher recall value for each phenotype could be achieved at the cost of a lower precision rate. Because AHE cases account for 0.6% of the sample, our method, in the best scenario, can correctly predict 20% of AHE cases, hence representing a substantial improvement over random guessing. For both AHE and HRS, the low AUC values do not necessarily convey poor performance; rather, they indicate that the imbalanced distributions make accurate phenotype predictions very difficult.

### Value of Latent Regulator to Predictive Power

We incorporate a latent regulator in the RNNs as an important novelty of the proposed method. We therefore specifically examined its value regarding our proposed method’s predictive utilities. Table 7 summarizes the model’s predictive power, with and without the latent regulator. The method is more effective for predicting AHE if it includes the latent regulator, as indicated by the 40% improvement in recall, 24% in AUC, 31% in precision, and 33% in the F-measure, compared with its application without the regulator. For HRS, incorporating the latent regulator leads to an improvement of 7% in AUC, 14% in precision, and 18% in F-measure. These comparative results confirm that the value of the latent regulator (latent parameter matrix) is relative to techniques that only rely on available patient data.

**Table 7:** Predictive performance of the proposed method, with versus without the latent regulator.

Performance measure	Our method without latent regulator			Our method (RNN-LR^a^)		
	Neither	AHE^b^	HRS^c^	Neither	AHE	HRS
Precision	0.97	0.16	0.07	0.98	0.21	0.08
Recall	0.89	0.30	0.26	0.85	0.42	0.40
F-measure	0.93	0.21	0.11	0.91	0.28	0.13
AUC^d^	0.58	0.66	0.60	0.66	0.82	0.64

^a^RNN-LR: recurrent neural network-latent regulator.

^b^AHE: acute hepatic encephalopathy.

^c^HRS: hepatorenal syndrome.

^d^AUC: area under the curve.

### Ablation Analysis

**Figure 8:**
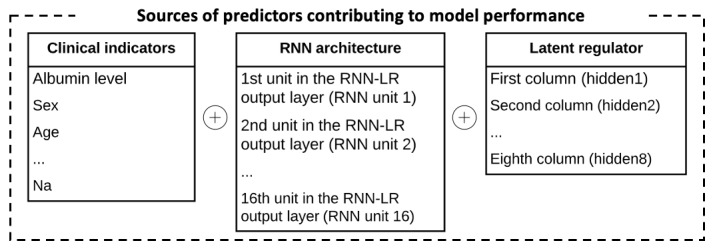
Different predictors in our method and their contributions to predictive power. RNN: recurrent neural network; RNN-LR: recurrent neural network-latent regulator; Na: potassium.

Furthermore, we analyzed the proposed deep learning–based method with deep SHAP [80] to reveal the elements that contribute more predictive power. In essence, SHAP follows a game theoretic approach to analyze the output of a predictive model and indicate the marginal contributions of different features to predictions [80]. We categorized the predictors of our RNN-LR method as clinical indicators (eg, patient’s age, sex, and albumin level), RNN architecture (data representations in different units of RNN-LR output), and latent regulator (vectors in the parameter matrix), as shown in Figure 8. Having specified the different types of predictors, we used deep SHAP to identify variables that contribute more to our proposed method’s ability to predict complication phenotypes accurately.

**Figure 9:**
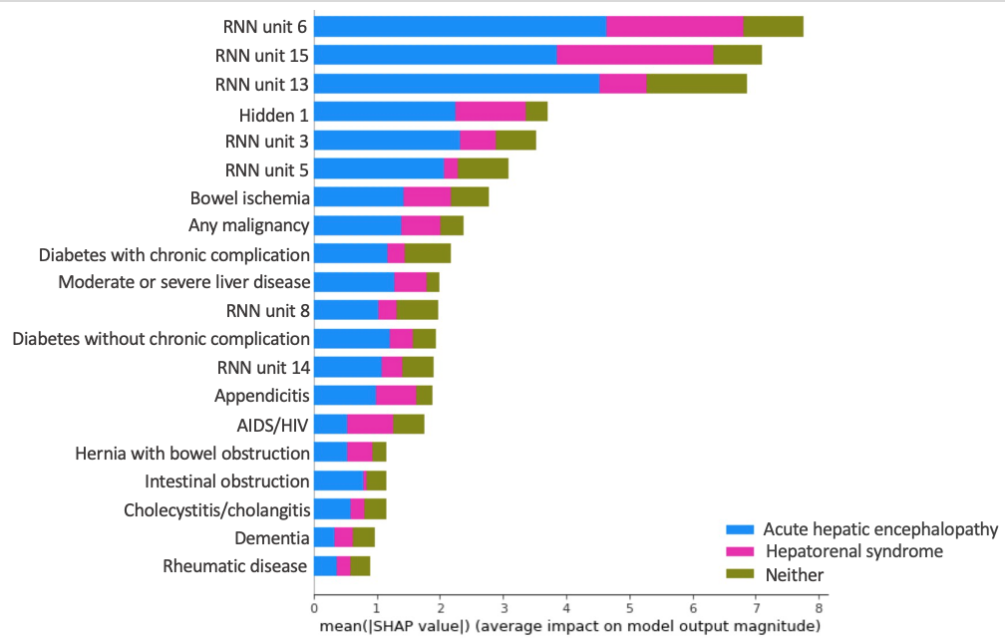
Mean SHAP values of different predictors in the proposed recurrent neural network-latent regulator method. RNN: recurrent neural network.

In Figure 9, we sort the different features according to the mean SHAP values, which are approximations of their contributions to the predictions. As shown in the figure, *RNN unit 6* contributes most to the method’s predictive utilities; that is, the sixth unit of the deep learning output provides the most valuable information to predict complication phenotypes. This feature is derived from diagnostic clinical outcomes (Table 1) and conveys the value of patient representation. Overall, the SHAP values indicate that 7 of the top 20 predictors relate to the RNN architecture, that is, the method’s architecture provides more important information to predict crucial complication phenotypes than clinical indicators or the latent regulator. Also, *hidden 1* refers to the first column vector of the latent regulator and is the fourth most important predictor, which confirms that the latent regulator contains important information for predicting AHE and HRS. Bowel ischemia and malignancy are two important clinical indicators for predicting crucial complication phenotypes.

### Summary

The evaluation results demonstrate the advantages of the proposed method (RNN-LR) over several prevalent techniques. Our method outperforms temporal MMCBR [10] because it employs a recurrent neural network to learn the underlying features of the patient’s condition and disease progression, rather than relying on available clinical data to perform clustering analyses. Time fusion CNN measures pairwise similarity in patient progression to predict complication phenotypes. In the presence of substantial missing values in patient data, as illustrated in our sample, pairwise similarity may not effectively capture clinical progression variations, which confines the predictive power of time fusion CNN [12]. Patient data, such as laboratory results, are gathered at different frequencies and time intervals. To address such temporal heterogeneities, T-LSTM learns patient representation from input (patient) data with different time intervals [11]. The proposed method instead imputes missing values in patient data to generate same-length input (data) sequences and achieves better predictive performance than T-LSTM. In particular, the use of a latent regulator, as an additional parameter matrix, to mitigate the information insufficiency constraint helps in capturing the underlying relationships of clinical factors to produce better predictions, together with the available patient clinical data. In summary, the proposed deep learning–based method addresses imbalanced outcome distributions in patient data and considers patient-level temporal heterogeneities to predict AHE and HRS by incorporating both a latent regulator and cost-sensitive analysis to extend back-propagation learning in deep neural networks.

## Discussion

### Principal Findings

This study offers several implications for health informatics and improved acute disease patient management. First, data insufficiencies represent a challenge to physicians’ patient care and management. This study highlights the promising use of a latent parameter matrix to alleviate this constraint by demonstrating its feasibility and clinical value in the prediction of crucial complication phenotypes. This latent parameter matrix can be modified or extended to accommodate other variables or hidden risk factors to more effectively predict important patient outcomes. Second, patient data are temporally heterogeneous, which creates another difficulty for clinically using EHRs and predictive analytics. Such heterogeneities can be addressed with effective missing data imputations that learn temporal feature representations from patient data to render increased predictive utilities. Unlike many existing techniques that overlook temporal heterogeneities or inconsistencies in patient data [56,81], we illustrate that an explicit incorporation of an effective representation for temporal heterogeneities can improve predictive performance. Third, imbalanced distributions of patient outcomes prevail in clinical scenarios, which creates an additional difficulty for leveraging predictive analytics in health care. Although only a small proportion of patients develop severe complication phenotypes, the outcomes can be harmful or even fatal. We demonstrate the value of cost-sensitive learning for an increased efficacy in crucial phenotype predictions (AHE or HRS). Effective patient representation, such as short-term temporal representations from limited observed patient data, and a latent regulator jointly enable patient information abstraction at multiple levels to predict complication phenotypes more accurately.

Our research also has important implications for clinical practice. Health care is going through a paradigmatic shift from reactive care to preventive care. Predicting important clinical events and patient outcomes, especially among patients with acute diseases, is critical to the quality of care and cost containment in patient management. The proposed method can be applied to develop advanced clinical decision support systems that assist physicians at the point of care. For example, a timely detection of patients who are likely to develop severe complications is critical but challenging. Through support by decision support systems enabled by the proposed method, physicians can identify at-risk patients and perform thorough monitoring and timely interventions to improve those patients’ outcomes and well-being. Our method can also benefit health care organizations in their resource planning and allocation. For example, effective phenotype predictions can help a hospital distinguish patients who are likely or not likely to develop serious complications, so their readmission risk or length of stay can be reduced. Such benefits have important implications for resource utilization efficiency and cost containment in patient care and management.

### Conclusions and Future Research

We have developed a deep learning–based method to predict crucial complication phenotypes of an acute disease. Furthermore, we have evaluated the proposed method and several prevalent benchmark techniques with a peritonitis data set by comparing their predictions of AHE and HRS. The empirical results reveal the advantageous predictive power of our method, which can address challenges pertaining to data insufficiency, temporal heterogeneity, and imbalanced outcome distributions. This study makes several contributions to the predictive analytics for an improved care and management of patients with acute diseases. First, we demonstrate the feasibility and clinical value of using a latent regulator to cope with insufficiencies in available patient data to improve phenotype predictions. The latent regulator, incorporated in the proposed method, can be expanded to model other external variables and hidden risk factors for predicting different complication phenotypes. Second, our proposed method incorporates missing data imputation and addresses temporal heterogeneities that exist in patient data, a fundamental challenge in using EHRs and predictive analytics for patient care and management. As we illustrate, temporal feature representation can be learned from patient data to provide increased predictive utilities. Third, imbalanced data prevail in clinical scenarios. Although only a relatively small proportion of patients develop severe complication phenotypes, the outcomes can be fatal. Toward that end, the proposed method reveals the value of cost-sensitive learning to address the data imbalance issue and demonstrates greater effectiveness to predict the minority class (eg, AHE and HRS), which is clinically important.

This study has several limitations that warrant continued research attention. First, the sample for the evaluation was relatively limited in size with respect to the disease category. Continued research should re-examine the proposed method with additional, diverse patient samples and different acute diseases. Second, we rely on domain experts to guide clinical feature extraction in this study. We acknowledge that some potentially important factors might be overlooked by domain experts. In addition, other complications may involve more complex risk factors, such as patient comorbidity, disease progression, and environmental factors. Thus, further research should explore how representation learning might identify features automatically from various patient clinical and behavioral data. Third, a predictive model’s ability to generate interpretable results is desirable and important; however, interpreting the proposed method’s predictions is difficult because its deep learning model maps input variables (eg, laboratory results, sex, age) to a numerical output variable through multiple layers. The complex structures make its prediction results difficult to interpret, unlike rule- or inductive decision tree–based techniques that can reveal interpretable relationships between input variables and the target variable. Ongoing research should explore *interpretable* computational methods built on explainable artificial intelligence. In a related sense, our method uses a latent regulator to account for observed disease progression and underlying mechanisms (eg, hidden disease patterns), so its processing and results cannot explain the underlying causes of the phenotypes. Continued efforts are needed to specify and test probable mechanisms and pathogeneses leading to crucial hepatic complications, as manifested by these phenotypes.

## Abbreviations

AHE: acute hepatic encephalopathy
AUC: area under the curve
CGRD: Chang Gung Research Database
CNN: convolutional neural network
EHR: electronic health record
HRS: hepatorenal syndrome
ICU: intensive care unit
MMCBR: multiple measurement case-based reasoning
NHIRD: National Health Insurance Research Database
RNN: recurrent neural network
RNN-LR: recurrent neural network-latent regulator
SHAP: Shapley Additive Explanations
SMOTEENN: Synthetic Minority Oversampling Technique–Edited Nearest Neighbors
T-LSTM: time aware long short-term memory
